# Early Detection of Local Tumor Progression after Irreversible Electroporation (IRE) of Hepatocellular Carcinoma Using Gd-EOB-DTPA-Based MR Imaging at 3T

**DOI:** 10.3390/cancers13071595

**Published:** 2021-03-30

**Authors:** Wolf Bäumler, Philipp Wiggermann, Lukas Lürken, Marco Dollinger, Christian Stroszczynski, Lukas P. Beyer, Andreas Schicho

**Affiliations:** 1 Department of Radiology, University Hospital Regensburg, 93042 Regensburg, Germany; wolf.baeumler@ukr.de (W.B.); lukas.luerken@ukr.de (L.L.); marco.dollinger@ukr.dev (M.D.); christian.stroszczynski@ukr.de (C.S.); 2 Department of Radiology and Nuclear Medicine, Städtisches Klinikum Braunschweig, 38114 Braunschweig, Germany; p.wiggermann@klinikum-braunschweig.de; 3 Department of Radiology, Klinikum Ernst von Bergmann, 14467 Potsdam, Germany; lukas.beyer@klinikumevb.de

**Keywords:** irreversible electroporation, hepatocellular carcinoma, early detection, magnetic resonance imaging, Gd-EOB-DTPA

## Abstract

**Simple Summary:**

Liver tumors like hepatocellular carcinoma (HCC) can be treated minimally invasive, e.g., by Irreversible Electroporation (IRE), which destroys the cancer. As it is possible that the tumor re-grows due to single tumor cells inadvertently not covered by the treatment, follow-up imaging of the liver is important for early detection of local tumor progression. As ablation leaves scarred tissue, recurrent tumor after IRE can appear vastly different than before treatment and thus can be hard to detect on MRI via classical imaging features. We here examined cases of local tumor progression after IRE of HCC and found distinct MR-imaging features helpful for the identification of re-grown viable tumor, namely T2 BLADE and diffusion weighted images (DWI) at the ablation zone border and T1 portal-venous and delayed phase post-contrast images in the center of the ablation zone. This knowledge will help in early detection and re-treatment of HCC for a prolonged survival.

**Abstract:**

This single-center retrospective study was conducted to improve the early detection of local tumor progression (LTP) after irreversible electroporation (IRE) of a hepatocellular carcinoma (HCC) using gadolinium ethoxybenzyl diethylenetriamine pentaacetic acid (Gd-EOB-DTPA)-based 3T MR imaging and to identify helpful signal characteristics by comparing 23 patients with and 60 patients without LTP. To identify the differences in the sensitivity of MRI sequences, the specificity, positive prediction value, negative prediction value (NPV) and diagnostic odds ratio were calculated. A chi-squared test, two-tailed student’s t-test and binary logistic regression model were used to detect distinct patient characteristics and variables for the prediction of LTP. LTP was mostly detected in the peripheral ablation zone (82.6%) within the first six months (87.0%). The central LTP ablation area presented more hypointensities in T1 p.v. (sensitivity: 95.0%; NPV: 90.0%) and in T1 d.p. (sensitivity: 100.0%; NPV: 100.0) while its peripheral part showed more hyperintensities in T2 BLADE (sensitivity: 95.5%; NPV: 80.0%) and in diffusion sequences (sensitivity: 90.0%). Liver cirrhosis seems to be an unfavorable prognosticator for LTP (*p* = 0.039). In conclusion, LTP mostly occurs in the peripheral ablation zone within six months after IRE. Despite often exhibiting atypical Gd-EOB-DTPA MR signal characteristics, T2 BLADE and diffusion sequences were helpful for their detection in the peripheral zone while T1 p.v. and T1 d.p. had the highest sensitivity in the central zone.

## 1. Introduction

Although surgical resection plays a significant role in the therapy of a hepatocellular carcinoma, many patients do not qualify for surgery because of the cancer spread, localization near or infiltration of critical structures or certain comorbidities [1]. In this patient group, percutaneous ablation methods have increasingly been implemented in the clinical routine in recent years. Most of the currently applied ablation techniques such as microwave ablation, radiofrequency ablation or cryoablation are based on thermal changes of the ablated tissue. In contrast, irreversible electroporation (IRE) represents a predominantly non-thermal ablative method. By causing cell death through the repeated application of high-voltage electrical impulses, which generate irreversible damage to the membranes of tumor cells [2], IRE offers significant benefits over thermal-based ablative methods especially concerning safety. While thermal ablation techniques mostly entail the risk of damaging adjacent structures [3,4], several studies have proven that IRE protects the architecture of hepatic structures such as large vessels and bile ducts in close proximity to the IRE ablation area [1,5,6,7,8]. IRE is of interest for clinical use particularly because of its safety characteristics and its high efficacy [9,10].

As IRE requires the placement of at least two and up to seven parallel electrodes around the target lesion and as target lesions in IRE are often located in proximity to critical anatomical structures, both the risk of a primary incomplete ablation as well as delayed local tumor progression from microscopically small residual tumors are of concern [11]. This is tackled by establishing sufficient safety margins in ablations and the early detection of local relapses. Both incomplete ablation and local tumor recurrence, which needs to be considered as a local tumor progression of non-ablated microresidue, have to be detected as early as possible to initiate further (re-)treatment.

Although magnetic resonance imaging (MRI) is considered to be the most effective imaging modality to detect hepatic tumors [12,13], it is still a challenge even for the experienced observer to differentiate between post-ablative tissue and local tumor progression. This is because the signal behavior of the ablation area is complex and changes over time, e.g., due to hemorrhagic transformation, the hemoglobin oxygenation state and heme oxidative denaturation to the ferric (Fe^3+^) form. Gadolinium ethoxybenzyl diethylenetriamine pentaacetic acid (Gd-EOB-DTPA) is a gadoxetic acid-based MRI contrast agent (Eovist^®^/Primovist^®^; Bayer Schering, Berlin, Germany) and is considered the most sensitive option for hepatocellular carcinoma (HCC) imaging especially in combination with a 3 Tesla scanner [14]. About 50% of the injected dose of Gd-EOB-DTPA is taken up via the organic anion transporter protein 1 and excreted by the biliary route in healthy hepatocytes [15], which enables the detection of an HCC as a non-enhancing lesion in a so-called delayed phase. So far, the literature provides no specific information whether Gd-EOB-DTPA-based MRI at 3 Tesla offers reliable criteria for the detection of local tumor progression after IRE of HCCs.

Thus, the aim of this study was to find imaging characteristics that help to improve the detection of a local tumor relapse after IRE of an HCC, which is the hallmark for initiation of early and successful treatment; a small tumor size and early treatment are two of the most important factors influencing survival in almost all types of cancer including HCCs [16,17].

## 2. Patients and Methods

### 2.1. Study Design, Patient Selection and Patient Characteristics

**Figure 1:**
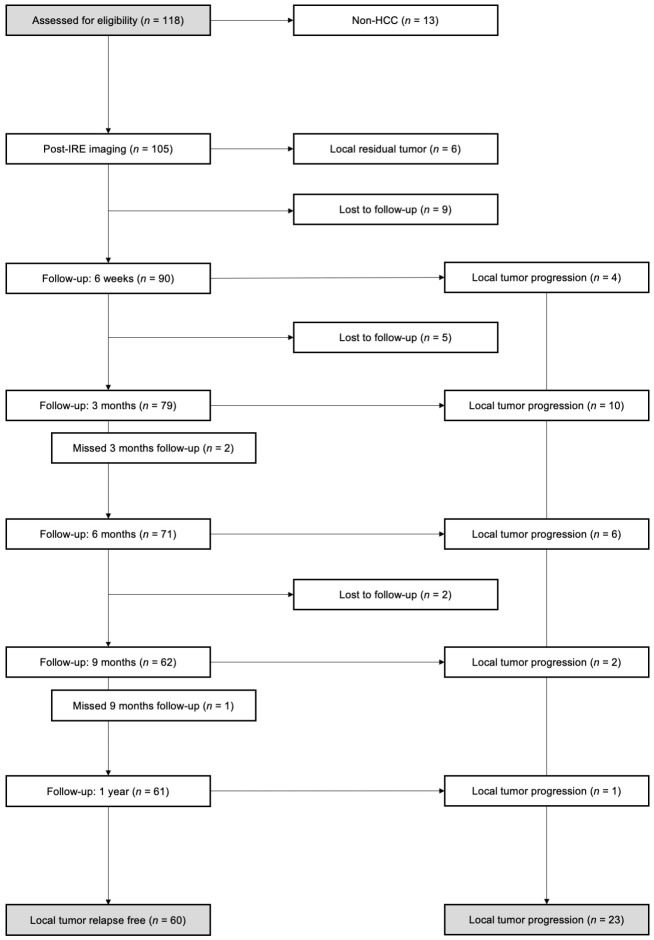
Number of patients during follow-up.

The study was conducted in accordance with the World Medical Association Declaration of Helsinki, the guidelines of relevant local authorities and legal regulations. It was approved by the local ethics committee (#18-1027-104). To determine characteristic changes in the MR imaging appearance of the ablation area in patients a with local tumor progression after percutaneous IRE of an HCC, the follow-up MR images of all IRE procedures performed at the University Hospital of Regensburg between December 2011 and August 2019 were retrospectively evaluated. Follow-up contrast-enhanced 3 Tesla (3T) MR imaging using Gd-EOB-DTPA including a 20 min delayed hepatobiliary phase was routinely performed at five defined timepoints after IRE. A first follow-up MRI was conducted on day one or two after ablation. Further follow-up MR imaging was performed six weeks, three months, six months, nine months and one year after ablation, according to our institution’s standard of practice. Patients with a local tumor progression formed one group (local tumor progression; LTP-group). The control group (relapse-free; RF-group) contained all patients without a local tumor progression within one year of follow-up (Figure 1). Thus, the following inclusion criteria were applied: (I) a histologically proven HCC, (II) an HCC treated by percutaneous IRE, (III) for patients of the LTP-group, a local tumor progression during the first year after IRE either histologically proven or unequivocal by imaging criteria, (IV) written informed consent for the acquisition of pre- and post-interventional contrast-enhanced 3T MR images using Gd-EOB-DTPA, the IRE ablation procedure itself and the anonymous use of the data for scientific purposes in accordance with our standard of practice and with the above-stated applicable regulations.

Additionally, the following exclusion criteria were defined: (I) a residual tumor, which had been detected in the immediate post-interventional MRI within 24 h after IRE, (II) the presence of a lesion in the post-interventional ablation area being suspicious for a local tumor progression of an HCC that was not confirmed histologically.

### 2.2. Irreversible Electroporation (IRE)

All irreversible electroporation procedures/ablations were performed percutaneously under computed tomographic (CT) fluoroscopy guidance and full anesthesia including deep muscle relaxation using the NanoKnife system (AngioDynamics, Latham, NY, USA). At the discretion of the interventionalists and technical feasibility, a biopsy of the LTP was taken immediately before reablation. The operator placed two to six monopolar 18-gauge IRE probes parallel to each other in or around the target tumor, depending on the size and position of the target lesion. The IRE parameters were as follows [18]: pulses per cycle, 70; pulse length, 90 µs; electric field, 1500 V/cm needle distance with individual adaption following the manufacturer’s instructions (range: 1000–3000 V). A test pulse of 270 V was delivered before the delivery of 90 therapeutic pulses (range: 70–100 pulses) to confirm sufficient conductivity. If the current between two electrodes exceeded 48 A (high-current condition), pulses were aborted to prevent heat induction.

### 2.3. Image Acquisition

MR imaging was performed with a clinical whole-body 3T system (MAGNETOM Skyra; Siemens, Erlangen, Germany). All patients were examined by contrast-enhanced MR scans using the hepatocyte-specific contrast agent gadolinium ethoxybenzyl diethylenetriamine pentaacetic acid (Gd-EOB-DTPA; Eovist^®^/Primovist^®^; Bayer Schering, Berlin, Germany). The contrast agent was applied by an intravenous bolus injection at a flow rate of 1 mL/s and flushed with 20 mL of NaCl 0.9%. The dose was calculated as 0.025 mmol/kg of body weight according to the manufacturer’s recommendations.

The MR protocol included the following sequences: T2 HASTE contrast-enhanced, T1 vibe3d fat suppressed unenhanced, T1 vibe3d fat suppressed contrast-enhanced arterial phase (T1 art.), T1 vibe3d fat suppressed contrast-enhanced portal venous phase (T1 p.v.), T2 BLADE fat suppressed contrast-enhanced, diffusion trace contrast-enhanced (*b*-value: 800 s/mm^2^) and T1 vibe3d fat suppressed contrast-enhanced delayed phase (T1 d.p.). Every sequence contained the entire liver and both subtracted and unsubtracted images were available and used for reading.

Two radiologists with five years and seven years of experience in liver imaging, respectively, examined the post-interventional MR images for the appearance of the ablation zone and the presence of a local tumor relapse by consensus reading. Criteria used were (1) a new nodular mass, (2) classic imaging features of an HCC including early (arterial) contrast-enhancement or portal venous and delayed phase wash-out and/or (3) divergent signal characteristics from the ablation zone and neighboring non-ablated liver tissue. The ablation area was classified into two parts, a central and a peripheral zone, which were evaluated separately from each other. The peripheral zone was defined as the distance measured from the rim of the ablation zone that corresponded with 10% of the zone’s maximum diameter. The remaining inner part of the ablation area was defined as its central zone. The signal intensity of the central and the peripheral zone in each available sequence was categorized in one of three groups: hypointense, isointense or hyperintense relative to the healthy liver parenchyma in the same sequence.

According to the International Working Group on Image-guided Tumor Ablation, the Interventional Oncology Sans Frontières Expert Panel, the Technology Assessment Committee of the Society of Interventional Radiology (SIR) and the Standard of Practice Committee of the Cardiovascular and Interventional Radiological Society of Europe (CIRSE), a local tumor progression (LTP) describes the appearance of tumor foci after at least one study has documented adequate ablation and an absence of viable tissue in the target tumor as well as in the surrounding ablation margin regardless of when tumor foci were discovered either early or late in the course of imaging follow-up [19]. While the term local tumor recurrence might be favorable in the clinical use, the term local tumor progression should be preferred as we have to assume that local recurrences are indeed the consequence of incomplete ablations despite no viable tissue being detectable in one or more post-ablation imaging studies.

### 2.4. Statistical Analysis

To identify potential differences between both patient groups, the biometric and tumor characteristics were compared by either a two-sided student’s t-test with alpha = 0.05 or a comparison of proportion using the N-1 chi-squared test. A comparison was made between the mean values of all MRI sequences in the LTP-group measured in the follow-up control in which the local tumor progression had been detected for the first time and the mean values of the RF-group, calculated from the data of the entire one year follow-up control (six weeks to one year after IRE). All collected data are presented as frequency counts and percentages.

The intrinsic test characteristics (sensitivity and specificity) and the performance in the selected population (positive and negative predictive values) were calculated according to standard formulas using a 2 × 2 contingency table as follows: sensitivity (true positive rate, TPR) = TP/P; specificity (true negative rate, TNR) = TN/N; positive prediction value (PPV) = TP/(TP + FP); negative prediction value (NPV) = TN/(TN + FN); diagnostic odds ratio (DOR) = LR + /LR−; positive likelihood ratio (LR+) = TPR/FPR; negative likelihood ratio (LR−) = FNR/TNR; false negative rate (FNR) = 1−TPR where TP = true positive, P = positive, TN = true negative, N = negative, FP = false positive, FN = false negative, FPR = false positive rate and FNR = false negative rate.

Higher signals were considered as indicative for an HCC for T2 BLADE fat suppressed contrast-enhanced, T2 HASTE contrast-enhanced, T1 vibe3d fat suppressed unenhanced, diffusion trace contrast-enhanced and T1 vibe3d fat suppressed contrast-enhanced arterial phases [20]. For the T1 vibe3d fat suppressed contrast-enhanced portal venous phase and the T1 vibe3d fat suppressed delayed phase, lower signals were considered as indicative of an HCC (“wash-out”). When compared with non-affected liver tissue, isointense signals were considered indeterminate for statistical calculations as this information does not contribute to clinical decision making. Concerning sensitivity, specificity, PPV and NPV, values ≥ 80.0% were considered relevant if a simultaneous DOR value ≥ 1.0 could be observed. The only exception was constituted by the T1-delayed phase sequence in the central part of the ablation area. In this case, no DOR value could be calculated.

To identify variables for the prediction of the local tumor progression of an HCC, a binary logistic regression model was used. Equal correlation of the binary response for individual patients was assumed, implying an exchangeable correlation structure. The variables analyzed were the presence of liver cirrhosis, the localization of the HCC (segment I–IV vs. segment V–VIII), the distance between the tumor and skin (≤60 cm vs. >60 cm), the number of IRE electrodes (≤3 vs. >3) and the localization of the HCC (subcapsular vs not subcapsular).

All statistical analyses were performed using Microsoft Excel (Microsoft Excel 2019, Redmond, Washington, DC, USA) and SPSS statistics (IBM SPSS Statistics, V. 25, Armonk, NY, USA). Values were either expressed as a percentage with or without a 95% confidence interval, maximum likelihood odds ratio estimators or as an arithmetic mean with a standard deviation. A *p*-value of ≤0.05 was considered statistically significant.

## 3. Results

In total, 83 patients (64 men and 19 women) fulfilled the inclusion criteria with 23 of them showing a local tumor progression (LTP) and 60 remaining tumor-free within the study-relevant 12-month time period serving as a control group. Each patient underwent irreversible electroporation of exactly one HCC lesion; this lesion was 2.1 ± 1.1 cm in the LTP-group and 2.3 ± 1.1 cm in the control group (*p* > 0.05). Further patient and disease characteristics are listed in Table 1; groups showed no statistically significant differences with an exception for the presence of liver cirrhosis (*p* = 0.023). In the binary logistic regression model, the presence of liver cirrhosis represented an unfavorable prognosticator of a local tumor progression of an HCC after IRE (*p* = 0.039, Table 2).

A total of 19 (82.6%) out of 23 patients had a local tumor progression in the peripheral ablation zone. In four patients (17.4%), an infiltration of both parts (the peripheral and the central ablation zone) was noted (Table 3). No sole central progression occurred.

In 43.5% (n = 10) of cases, a local tumor progression was detected three months after IRE and in 87.0% (n = 20) of all cases the tumor progression was noted within the first six months after IRE. Table 3 summarizes the different points of time of the local tumor progression.

The cumulative local tumor progression after MRI-verified primary ablation success in 99 out of 105 patients (94.3%) was 4/99 = 4.0%, 14/99 = 14.1%, 20/99 = 20.2%, 22/99 = 22.2% and 23/99 = 23.2% after six weeks, three months, six months, nine months and one year, respectively. The calculated MRI-verified three-month local progression-free rate was 69/105 = 65.7%, respectively, but 14/105 = 13.3% were lost to follow-up in this period.

On average, the mean diameter of the tumor relapse was 0.3 ± 0.1 cm on detection. A total of 14 out of 23 LTPs were histologically proven by a biopsy (60.7%).

**Figure 2:**
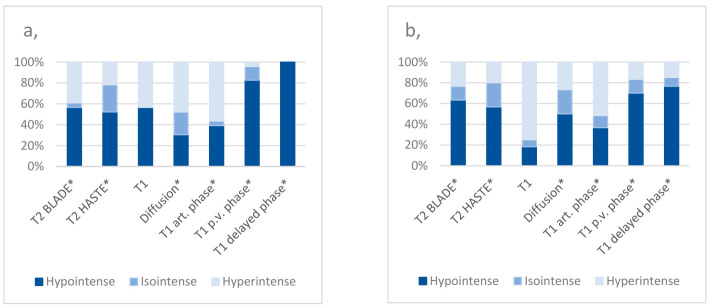
Proportions of signal characteristics in the central ablation zone of patients with a local tumor progression after IRE of an HCC (**a**) and of the reference group (**b**) * = contrast-enhanced; art. phase = arterial phase; p.v. phase = portal venous phase.

Comparing the MR imaging signal characteristics of the central ablation zone between the groups, T1 p.v. had a sensitivity of 95% and a negative predictive value of 90% for the detection of local tumor progressions. T1 d.p. had a sensitivity of 100% and negative predictive value of 100%, respectively. The diffusion sequence had a negative predictive value of 81.08% (Table 4; Figure 2; Appendix A).

**Figure 3:**
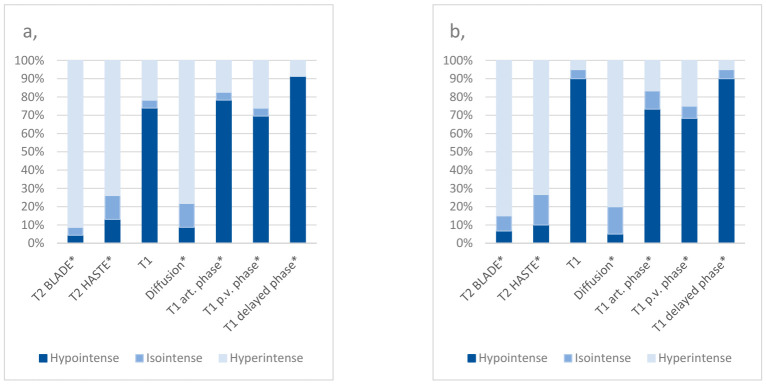
Proportions of signal characteristics in the peripheral ablation zone of patients suffering from a local tumor progression after IRE of an HCC (**a**) and of the reference group (**b**) * = contrast-enhanced; art. phase = arterial phase; p.v. phase = portal venous phase.

Regarding the peripheral ablation zone, the highest sensitivity (95.45%) and negative predictive value (80%) for the detection of local tumor progressions was noted for the T2 BLADE sequence. T2 HASTE showed a sensitivity of 85.0% and a diffusion sequence of 90%, respectively. T1 non-enhanced showed a specificity of 94.74% and T1 art. 81.48%, respectively. All calculated values are presented in detail inTable 5 and Figure 3.

As expected, the size of ablation defects showed an involution over time from 5.6 ± 1.3 cm in the LTP-group and 5.5 ± 1.5 cm in the reference group to 3.5 ± 1.1 cm for the LTP-group at six weeks after IRE and 3.4 ± 1.8 for the reference group, respectively (Table 6).

## 4. Discussion

Representing a minimally invasive option in the therapy of HCCs, the importance of IRE has increased during recent years. Although several reports have been published concerning the safety and efficacy of IRE [23,24,25,26], little is known about how and when to detect local tumor progressions to enable early re-treatment and halt progress. For example, Rimola et al. suggest post-interventional Gd-EOB-DTPA MRI including a hepatobiliary-phase and DWI (diffusion weighted imaging) sequences to detect early HCC progression [27]. Unfortunately, their study does not contain a differentiation between patients who were treated with surgery or several ablation techniques. To the best of our knowledge, this is the first study addressing these questions using standard of care Gd-EOB-DTPA-based MR imaging at 3 Tesla.

Overall, six out of 105 patients (5.7%) had an incomplete ablation detected on the immediate post-IRE MRI. All six underwent successful reablation but were not covered further in our study after drop-out per our protocol (Figure 1). Incomplete ablation is any residual viable tumor after ablation. A newly detectable viable tumor after adequate ablation and an absence of viable tissue in the target tumor and surrounding ablation margin documented by imaging criteria is termed tumor progression as these cases most likely resemble residual untreated microscopic tumors [19]. While technically both incomplete ablations and tumor progressions feature a residual viable tumor, our clinical work was limited by the imaging resolution and visibility of these tumor remnants. This phenomenon or limitation perfectly parallels the well-known “R” status in tumor surgery. Minimally invasive oncological therapies such as IRE or other ablation techniques have the inherent shortcoming of tissue destruction, which forecloses the pathological work-up of the border that serves as a quality check. As a consequence, post-ablation imaging and close controls are of the utmost importance as we cannot rely on a microscopically validated R0 status.

In 82.6% (*n* = 19) of all patients suffering from a local tumor progression after IRE of an HCC, the tumor progression was detected in the periphery of the ablation area. In all of the other patients of the LTP-group (four patients; 17.4%) a local tumor progression was observed in the periphery and simultaneously in the center of the ablation zone (Table 2) although which part of the ablation zone housed the origin of the tumor could not be differentiated. Thus, it needed to be taken into consideration that all progressions actually arose in the peripheral zone and the here-mentioned cases represented a secondary tumor invasion of the central zone. Without doubt, the findings indicated that the peripheral part of the ablation area was affected more often by a local tumor progression than its center, which was in line with several other studies that have pointed to the relevance of a sufficient safety margin in ablations paralleling the well-known safety margin of surgical resection. An incomplete ablation of the peripheral ablation zone has to be contemplated as a potential reason for a local tumor progression. This hypothesis coincided with the results of Padia et al. [28] who assessed the post-IRE ablation areas of patients with an HCC by evaluating MR imaging. The authors described a temporary enhancement in the periphery of the ablation zone, which had been observed one day after IRE but had not been observable in further follow-up controls. Padia et al. suggested that this phenomenon might be caused by so called “reversible electroporation” [28]. This effect could lead to an incomplete ablation of peripheral tumor tissue and, in a few cases, facilitating the occurrence of a local tumor progression of a residual untreated microscopic tumor [19]. To verify this assumption, a histopathological correlation, which would have had to be performed immediately after IRE but was not feasible in a daily routine, would have been necessary.

In 87% (*n* = 20) of all patients of the LTP-group the tumor relapse was noted within the first six months after IRE (Table 3) while the peak was observed three months after the intervention (43.5%; *n* = 10). This outcome was consistent with the results of Kalra et al. who treated 21 HCCs with IRE and reported a median time to a local tumor progression of four months in five cases [29]. On the contrary, Sutter et al. described a median time to a local tumor progression of nine months in 15 cases after the complete ablation of 69 HCCs by IRE [30].

Taking all of the mentioned results into consideration, a closely performed follow-up control after IRE of hepatocellular carcinomas seems to be indispensable especially during the early period including at least the first six post-interventional months as a lack of follow-up could lead to a missed detection of an early local tumor progression. For the evaluation of a late-occurring local tumor progression, long-term studies consisting of a larger study population are necessary, but several cohorts have shown that a local tumor progression remains a concern even in the delayed timeframe ≥ 6 months after the initial successful IRE of the target lesion [18,23,31,32,33].

Being considered to be the most effective imaging modality to detect hepatic malignancies [12,13], MRI is currently the most commonly applied method for the follow-up control after IRE in the clinical daily routine. Nevertheless, the MRI appearance of the ablation area after IRE frequently differs greatly, often representing a diagnostic challenge for the observer. To detect the potential characteristic signal intensities of a local tumor progression after IRE of an HCC in Gd-EOB-DTPA-based MR imaging, the authors aimed to identify certain MRI sequences providing a minimum risk of missing a local tumor progression during follow-up. Therefore, the focus was placed on MRI sequences that showed the highest sensitivity and negative prediction values (NPV) in comparison with the reference group. It is of notable interest that the sequences considered the best, based on our results, differed for the central and peripheral ablation zone; in the center, the T1 d.p. and T1 p.v. sequences were most beneficial while in the peripheral zone, T2 BLADE and diffusion sequences were (statistically) more favorable in our cohort. One limitation of this study worth mentioning here is the small number of central tumor progressions.

**Figure 4:**
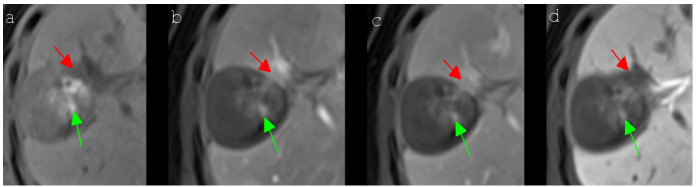
A 64-year-old woman with a local tumor progression in the peripheral ablation zone (segment V/VIII) six months after IRE of an HCC, which presented a typical signal behavior in Gd-EOB-DTPA-based MR imaging. The HCC showed a hypointense signal (red arrow) in the unenhanced T1 MR image (**a**), an obvious hyperintense signal (red arrow) in the contrast-enhanced T1 arterial phase MR image (**b**), a slight wash-out (red arrow) in the contrast-enhanced T1 portal venous phase MR image (**c**) and a hypointense signal (red arrow) in the contrast-enhanced T1 delayed phase MR image (**d**). In addition to this, the central part of the ablation area showed several hyperintense spots (green arrow), which could be detected in all presented MRI sequences (**a**–**d**), being caused by post-interventional bleeding.

**Figure 5:**
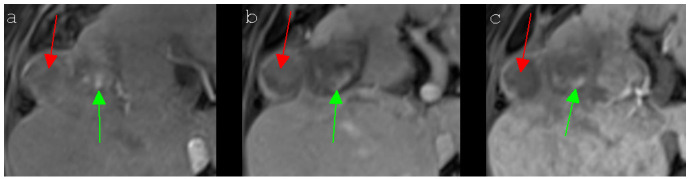
A 67-year-old man with a local tumor progression in the peripheral ablation zone (segment VIII/Iva) six months after IRE of an HCC, which presented atypical signal behavior in Gd-EOB-DTPA-based MR imaging. The nodular, exophytic HCC (red arrow) showed a slight peripheral enhancement and a central hypointensity in the contrast-enhanced T1 arterial phase MR image (**a**), the typically known hypervascularization was lacking, however. The signal intensities of the HCC remained unchanged during the contrast-enhanced T1 portal venous phase (**b**) and the contrast-enhanced T1 delayed phase MR image (**c**). A typical “wash-out” could not be observed. The central part of the ablation area showed several hyperintense spots (green arrow), which could be detected in all presented MRI sequences (**a**–**c**), attributable to post-interventional hemorrhagic transformation.

In addition, the detection of local tumor progressions is complicated by the fact that the observed MRI signal characteristic of recurrent tumors after IRE often differ from the classic MRI appearance of HCCs in Gd-EOB-DTPA-based MR imaging [34]. Presumably, this is caused by the interfering signal behavior of the post-ablative tissue. Figure 4 illustrates the signal behavior of a local tumor progression of an HCC being identical with the typical MRI appearance of an HCC as described in the literature [34]. In contrast, Figure 5 shows a tumor relapse after IRE of an HCC presenting atypical signal behavior, easily missed by the non-experienced reader [35]. Taking everything into consideration, the current study indicated that the detection of a local tumor progression in Gd-EOB-DTPA-based MR imaging after IRE of an HCC remains challenging as the observer cannot focus solely on the signal behavior of the ablation area. Although a few significant changes in the MRI sequences of the LTP-group could be identified, the signal intensities of patients with a local tumor progression after IRE of an HCC did not show classic HCC characteristics to ensure a reliable diagnosis. Therefore, it was even more important for the observer to additionally focus on the configuration of the ablation area in order to detect suspicious inhomogeneous parts or even progressively growing tissue (Figure 5). Knowing which MRI sequences are most conclusive is of great importance for the individual patient possibly requiring re-treatment. In cases of doubt, additional imaging techniques such as contrast-enhanced ultrasound or computed tomography can be used. If in doubt of indeterminate MR imaging characteristics, the radiologist can recommend a biopsy, which is also the standard of care in our institution. Additionally, closely performed MR imaging follow-up controls of the ablated area should be conducted.

The baseline characteristics of patients with and without a local tumor progression and the binary logistic regression model indicated that there was a significant difference in the presence of liver cirrhosis in patients with and without a local tumor progression (*p* = 0.023, Table 1) and that liver cirrhosis may increase the risk of a local tumor progression of an HCC after IRE (*p* = 0.039, Table 2). These findings were consistent with the findings of other authors who identified liver cirrhosis as a risk factor for the development of HCCs [36,37] and underlined the importance of its routine evaluation in patients suffering from an HCC even after being treated with IRE.

The present study had several limitations; the first was the retrospective and non-blinded design of the study. The second was the small cohort size owing to the single-center design. Third, there was an inconsistency between the LTP-group and the reference group concerning the number of patients. Fourth, both patient groups consisted of a heterogenous patient population concerning sex and age but exhibited no statistically significant difference in those baseline characteristics. Nonetheless, this study provided the largest cohort addressing the highly relevant topic of local tumor progression detection after IRE of HCCs.

## 5. Conclusions

Our study revealed helpful insights for the early detection of local tumor progressions after IRE of HCCs. First, most cases of a local tumor progression could be found within three to six months post-intervention, respectively. Thus, follow-up using Gd-EOB-DTPA-enhanced 3T MRI at three and six months after IRE is recommendable. Second, most progressions were located in the peripheral ablation zone. This finding supports the importance of sufficient ablation oversizing in IRE and calls for an especially careful evaluation of the periphery in follow-up imaging. Third, a few contrast-enhanced MRI sequences (T2 BLADE and diffusion sequences for the peripheral zone; T1 p.v. and T1 d.p. for the center) had the highest sensitivity and NPV for the detection of tumor progressions; however, a few HCC progressions showed atypical signal characteristics. Therefore, it is of utmost importance to evaluate each and every MRI sequence available. Fourth, liver cirrhosis seemed to be an unfavorable prognosticator for the development of local HCC progressions.

## Figures and Tables

**Table 1:** Patient and disease characteristics.

Characteristics	Patients with a Local Tumor Progression	Reference Group	*p*-Value
Number of patients	23	60	
Age (y)			
Mean ± SD	67.8 ± 11.3	67.6 ± 11.2	0.460 ^2^
Median (IQR)	68.0 (16.0)	68.0 (17.0)	
Range	48–85	36–84	
Sex, *n* (%)			
Male	17 (73.9)	47 (78.3)	0.671 ^1^
Female	6 (26.1)	13 (21.7)	
Patients with liver cirrhosis, *n* (%)	9 (39.1)	40 (66.7)	0.023 *^1^
Tumor localization, *n* (%)			
Segment I	0 (0.0)	3 (5.0)	0.278 ^1^
Segment II	3 (13.0)	6 (10.0)	0.696 ^1^
Segment III	4 (17.4)	5 (8.4)	0.242 ^1^
Segment IVa	2 (8.7)	5 (8.4)	0.965 ^1^
Segment IVb	3 (13.0)	7 (11.6)	0.861 ^1^
Segment V	4 (17.4)	8 (13.3)	0.636 ^1^
Segment VI	2 (8.7)	7 (11.6)	0.705 ^1^
Segment VII	0 (0.0)	5 (8.4)	0.154 ^1^
Segment VIII	5 (21.8)	14 (23.3)	0.885 ^1^
Tumor diameter (cm), mean ± SD	2.1 ± 1.1	2.3 ± 1.1	0.263 ^2^
Tumor diameter (cm), median (IQR)	19.0 (10.0)	20.0 (9.0)	
Subcapsular localization of the tumor, *n* (%)	14 (60.9)	32 (53.3)	0.535 ^1^
Distance between tumor and skin (cm), mean ± SD	66.4 ± 23.1	73.1 ± 23.8	0.228 ^2^
Distance between tumor and skin (cm), median (IQR)	65.0 (39.0)	70.0 (31.0)	
Tumors associated with vascular structures, *n* (%)	13 (56.5)	43 (71.7)	0.189 ^1^
Number of IRE electrodes, mean ± SD	4.7 ± 1.2	4.7 ± 1.0	0.94 ^2^
Number of IRE electrodes, median (IQR)	5.0 (2.0)	5.0 (2.0)	

SD = standard deviation, IQR = interquartile range, * significant difference (*p* < 0.05), ^1^ using the “N-1” chi-squared test as recommended by Campbell (2007) and Richardson (2011) [21]. ^2^ using a two-tailed student’s *t*-test for independent samples (IBM SPSS Statistics, version 25, Armonk, New York, USA) [22].

**Table 2:** Results of a binary logistic regression model predicting the local tumor progression of a hepatocellular carcinoma after irreversible electroporation.

Variable	OR (95% CI)	*p*-Value
Liver cirrhosis: no vs. yes	0.34 (0.12–0.94)	0.039 *
Localization of the HCC: Segment I–IV vs. V–VIII	0.77 (0.26–2.28)	0.641
Distance between tumor and skin: ≤60 cm vs. >60 cm	0.70 (0.24–2.01)	0.503
Number of IRE electrodes: ≤3 vs. >3	0.99 (0.25–3.90)	0.991
Localization of the HCC: subcapsular vs. not subcapsular	1.45 (0.50–4.21)	0.496

HCC, hepatocellular carcinoma; OR, odds ratio; CI, confidence interval. * significant difference (*p* < 0.05).

**Table 3:** Distribution of the local tumor progression after IRE of an HCC.

Localization of Local Tumor Progression	Number of Patients, *n* (%)
Central ablation area	0 (0.0)
Central and peripheral ablation area	4 (17.4)
Peripheral ablation area	19 (82.6)
**Time of First Observation of Local Tumor Progression**	
Six weeks after IRE	4 (17.4)
Three months after IRE	10 (43.5)
Six months after IRE	6 (26.1)
Nine months after IRE	2 (8.7)
One year after IRE	1 (4.3)

IRE = irreversible electroporation; HCC = hepatocellular carcinoma.

**Table 4:** Diagnostic accuracy of MRI signal characteristics for the local tumor progression in the central part of the ablation area after IRE of HCC.

MRI Sequence	Sensitivity (95% CI)	Specificity (95% CI)	PPV (95% CI)	NPV (95% CI)	DOR
T2 BLADE	40.91 (20.71–63.65)	73.08 (58.98–84.43)	39.13 (19.71–61.46)	74.51 (60.37–85.67)	1.877
T2 HASTE *	29.41 (10.31–55.96)	53.85 (33.37–73.41)	29.41 (10.31–55.96)	53.85 (33.37–73.41)	0.486
T1	43.48 (23.19–65.51)	19.64 (10.23–32.43)	18.18 (9.08–30.9)	45.83 (25.55–67.18)	0.188
Diffusion *	61.11 (35.75–82.7)	65.22 (49.75–78.65)	40.74 (22.39–61.2)	81.08 (64.84–92.04)	2.948
T1 art. *	59.09 (36.35–79.29)	41.51 (28.14–55.87)	29.55 (16.76–45.2)	70.79 (51.96–85.78)	1.024
T1 p.v. *	95.00 (75.13–99.87)	17.65 (8.4–30.87)	31.15 (19.9–44.29)	90.00 (55.5–99.75)	4.078
T1 d.p. *	100.00 (85.18–100.0)	16.36 (7.77–28.8)	33.33 (22.44–45.71)	100.00 (66.37–100.0)	-/- ^1^

PPV = positive prediction value; NPV = negative prediction value; DOR = diagnostic odds ratio; CI = confidence interval; * = contrast-enhanced; art. = arterial phase; p.v. = portal venous phase; d.p. = delayed phase; ^1^ = unable to calculate.

**Table 5:** Diagnostic accuracy of MRI signal characteristics for a local tumor progression in the peripheral part of the ablation area after IRE of an HCC.

MRI Sequence	Sensitivity	Specificity	PPV	NPV	DOR
	(95% CI)	(95% CI)	(95% CI)	(95% CI)	
T2 BLADE	95.45	7.27	29.17	80.00	1.646
	(77.16–99.88)	(2.02–17.59)	(19.05–41.07)	(28.36–99.49)	
T2 HASTE *	85.00	12.00	aat27.87	66.67	0.773
	(62.11–96.79)	(4.53–24.31)	(17.15–40.83)	(29.93–92.51)	
T1	22.73	94.74	62.50	76.06	5.292
	(7.82–45.37)	(85.38–98.9)	(24.49–91.48)	(64.46–85.39)	
Diffusion *	90	5.88	27.27	60	0.562
	(68.3–98.77)	(1.23–16.24)	(17.03–39.64)	(14.66–94.73)	
T1 art. *	18.18	81.48	28.57	70.97	0.978
	(5.19–40.28)	(68.57–90.75)	(8.39–58.1)	(58.05–81.8)	
T1 p.v. *	72.73	26.79	28.07	71.43	0.975
	(49.78–89.27)	(15.83–40.3)	(16.97–41.54)	(47.82–88.72)	
T1 d.p. *	91.3	5.26	28	60	0.583
	(71.96–98.93)	(1.1–14.62)	(18.24–39.56)	(14.66–94.73)	

PPV = positive prediction value; NPV = negative prediction value; DOR = diagnostic odds ratio; CI = confidence interval; * = contrast-enhanced; art. = arterial phase; p.v. = portal venous phase; d.p. = delayed phase.

**Table 6:** Diameter of the ablation area during the follow-up.

Point of Time	Patients with a Local Tumor Progression		Reference Group	
	Diameter of the Ablation Area (cm)		Diameter of the Ablation Area (cm)	
	Mean ± SD	Median (IQR)	Mean ± SD	Median (IQR)
Six weeks after IRE	5.6 ± 1.3	5.6 (1.2)	5.5 ± 1.5	5.3 (1.1)
Three months after IRE	4.5 ± 1.3	4.2 (1.1)	4.3 ± 1.5	4.1 (1.2)
Six months after IRE	4.3 ± 1.1	4.2 (1.1)	3.6 ± 1.6	3.6 (1.0)
Nine months after IRE	3.8 ± 1.2	3.9 (1.0)	3.5 ± 1.7	3.4 (1.0)
One year after IRE	3.5 ± 1.1	3.5 (1.0)	3.4 ± 1.8	3.2 (1.1)

SD = standard deviation; IQR = interquartile range.

## Data Availability

The datasets generated during and/or analyzed during the current study are available from the corresponding author on reasonable request.

## Abbreviations

BLADE	Proprietary name for periodically overlapping parallel lines with enhanced reconstruction (PROPELLER) from Siemens Healthcare
CI	Confidence interval
CIRSE	Cardiovascular and Interventional Radiological Society of Europe
DOR DWI	Diagnostic odds ratio Diffusion weighted imaging
FN	False negative
FNR	False negative rate
FP	False positive
FPR	False positive rate
Gd-EOB-DTPA	Gadoxetate-Disodium-Ethoxybenzyl-Diethylenetriamine-pentaacetic-acid (C_23_H_28_GdN_3_Na_2_O_11_; Eovist^®^/Primovist^®^)
HASTE	Half-Fourier acquisition single-shot turbo-spin echo
HCC	Hepatocellular carcinoma
IRE	Irreversible electroporation
LR–	Negative likelihood ratio
LR+	Positive likelihood ratio
LTP	Local tumor progression
MRI	Magnetic resonance imaging
MWA	Microwave ablation
N	Negative
NPV	Negative prediction value
P	Positive
PPV	Positive prediction value
RF	Relapse-free/control
SD	Standard deviation
SIR	Society of Interventional Radiology
TN	True negative
TNR	True negative rate
TP	True positive
TPR	True positive rate
T1 d.p.	T1 delayed phase
T1 p.v.	T1 portal venous

## Appendix A

A total of six patients were identified as having a residual tumor after IRE. These HCCs showed the following signal characteristics (Table A1) with typical T1 arterial phase contrast enhancement in five out of six cases (83.3%) and typical T1 delayed phase wash-out in five out of six cases (83.3%). T2 BLADE and T2 HASTE signal hyperintensity were the second most frequent characteristics observed with four out of six cases each (66.7%).

**Table A1:** Distribution of MRI signal characteristics in six patients with residual tumor tissue after IRE of an HCC.

MRI Sequence	Signal Intensity		
	Hypointense	Isointense	Hyperintense
T2 BLADE *	0	2	4
T2 HASTE *	0	1	5
T1	1	3	2
Diffusion	0	3	3
T1 arterial phase *	0	1	5
T1 portal venous phase *	2	4	0
T1 delayed phase *	5	1	0

* = contrast-enhanced; IRE = irreversible electroporation; HCC = hepatocellular carcinoma.
